# Peel of Traditional Apple Varieties as a Great Source of Bioactive Compounds: Extraction by Micro-Matrix Solid-Phase Dispersion

**DOI:** 10.3390/foods9010080

**Published:** 2020-01-11

**Authors:** Ante Lončarić, Katarina Matanović, Perla Ferrer, Tihomir Kovač, Bojan Šarkanj, Martina Skendrović Babojelić, Marta Lores

**Affiliations:** 1 Faculty of Food Technology Osijek, Josip Juraj Strossmayer University of Osijek, Franje Kuhača 20, HR 31000 Osijek, Croatia; kmatanovic@ptfos.hr (K.M.); tihomir.kovac@ptfos.hr (T.K.); 2 Faculty of Chemistry, University of Santiago de Compostela, Campus Vida, E-15782 Santiago de Compostela, Spain; perla.ferrer.espinilla@usc.es (P.F.); marta.lores@usc.es (M.L.); 3 Department of Food Technology, University Centre Koprivnica, University North, Trg dr. Žarka Dolinara 1, 48000 Koprivnica, Croatia; bsarkanj@unin.hr; 4 Faculty of Agriculture, University of Zagreb, Svetošimunska 25, 10000 Zagreb, Croatia; mskendrovic@agr.hr

**Keywords:** polyphenols, matrix solid-phase dispersion, apple peel, experimental design

## Abstract

Micro matrix solid phase dispersion (micro-MSPD) was optimized by response surface methodology for the extraction of polyphenols from the peel of twelve traditional and eight commercial apple varieties grown in Croatia. The optimized micro-MSPD procedure includes the use of 0.2 g of sample, 0.8 g of dispersant, a 57% solution of methanol in water as the solvent and 5 mL of extract volume. The total polyphenolic index (TPI) and antioxidant activity (AA) were measured by spectrophotometric assays. Eighteen polyphenolic compounds were identified in all investigated apples by HPLC-DAD and LC-(ESI)-MS. The peel of traditional apple varieties had higher contents of all investigated polyphenols. Calculated relative contribution of polyphenol groups indicated non-flavonoids (28.6%) and flavanols (46.2%) as the major contributors to the total polyphenolic content in traditional and commercial apple varieties, respectively. The most abundant polyphenol in traditional apple peel was chlorogenic acid, procyanidin B2 and epicatechin (1143 ± 755 µg/g dw, 954 ± 343 µg/g dw and 560 ± 362 µg/g dw, respectively). The peel of varieties ‘Apistar’, ‘Bobovac’ and ‘Božićnica’ could be highlighted as an important source of polyphenols.

## 1. Introduction

Recently, there has been a growing interest in the preservation of traditional apple varieties, which can still be found in individual orchards. Due to favorable differences in their adaptive characteristics that developed through many years, many of these traditional varieties should be preserved for the modern fruit-culture, such as the shape of their fruit, their taste, peel and pulp colors and their higher resistance to diseases [1,2]. In Croatia, many of these varieties still exist in locations where they have adapted well to the environmental and geographic features, which make them desirable for breeding. Fruit from these varieties is often commercially unattractive, due to undesirable organoleptic properties. Fortunately, in Western countries, there is a change of the concept of food, where consumers in addition to organoleptic quality require products with nutraceutical properties that make them beneficial for human health [3]. Apples are generally considered “healthy food”; one of the most important features of apples, the one that makes them interesting for researchers, is their polyphenol content, especially flavan-3-ols, phenolic acids, flavonols, dihydrochalcones and anthocyanins [1,2,4,5,6]. These compounds are studied intensively in epidemiological research, because they have a positive impact on human health through the prevention of cardiovascular diseases and cancer, the regulation of plasma cholesterol metabolism, antiviral properties, the inhibition of *Helicobacter pylori* growth and staphylococcal enterotoxin A toxicity [7,8,9]. It has been suggested that traditional apples contain higher amounts of polyphenols when compared to commercial ones [2,10,11,12,13,14,15]. In addition, it is well known that apple peel, as a by-product of apple processing, contains a higher polyphenolic content and antioxidant capacity than apple flesh [2,16,17].

Currently, the extraction of phenolic compounds from fruit by-products attracts great attention. When it comes to the extraction of polyphenols from apple peel, it mostly includes solvent extraction, ultrasound assisted extraction [13,18], microwave, supercritical CO_2_ extraction and other treatments [19]. One of the newest methods applied for the extraction of polyphenols is matrix solid-phase dispersion (MSPD) extraction. Matrix solid-phase dispersion was invented in 1989 and patented by Barker in 1993 [20]. Since then, it has found an important place among the preparation techniques applied in the analysis of solid, semi-solid and viscous samples [21]. MSPD extraction is based on blending the sample matrix with solid support in a mortar, after which the obtained mixture is used as pre-column packing, from which analytes are eluted with a suitable solvent. The main advantage of MSPD over the classical extraction procedures is performing extraction and clean up at the same time [20]. The sample amount in the classical MSPD protocol is 0.5 g; this can be significantly reduced by MSPD miniaturization, which consequently reduces the consumption of sorbent, solvent and the preparation time [22,23]. MSPD is used for the extraction and isolation of polyphenols from pomegranate peel [24], Lamiaceae family [25], grape marc [21], blackcurrant powder [26], Radix Astragali [27], etc. To the best of our knowledge, the use of matrix solid-phase dispersion for the extraction of polyphenols from apple peel has not been considered at this moment. In studies dealing with apples, MSPD was mainly used for the determination of pesticides and mycotoxins in apples and apple-based products [28].

Therefore, the aim of this study was the recovery of polyphenols from the peel of twelve traditional and eight commercial apple varieties grown in Croatia using MSPD extraction. The reason for using MSPD extraction for the recovery of polyphenols from apple peels is providing insight into new extraction possibilities for recovering polyphenols from apple peel and thus providing a perspective for scaling-up the process. In addition, this study will enable the recognition of traditional apple varieties as a valuable source of bioactive compounds. New knowledge in traditional apple varieties can help with the diversification of the apple market, preventing the potential disappearance of these cultivars and enabling the preservation of apple biodiversity.

## 2. Materials and Methods

### 2.1. Chemicals

The material used as the dispersant phase was washed sea sand, 200–300 μm Scharlau (Chemie S.A., Barcelona, Spain), the extraction solvent was LC-MS grade methanol supplied by Scharlau (Chemie S.A., Barcelona, Spain), the ultrapure water was produced in the laboratory with a Milli-Q gradient system (Millipore, Bedford, MA, USA) and the formic acid was supplied by Merck (Darmstadt, Germany). Folin and Ciocalteau phenol reagent obtained from Sigma–Aldrich and sodium carbonate (Na_2_CO_3_, Panreac, Barcelona, Spain) were used for the determination of the total polyphenolic index. The 2,2-diphenyl-1-picrylhydrazyl (DPPH) and 6-hydroxy-2,5,7,8-tetramethylchroman-2-carboxylic acid (Trolox^®^) were purchased from Sigma–Aldrich (Chemie Check GmbH, Steinheim, Germany) and used for the determination of the scavenging activity of the apple peel extracts.

Individual standard stock solutions (2000–8000 µg/mL) were prepared in methanol from pure polyphenols, procyanidin A2, B1, B2, catechin, epicatechin, chlorogenic acid, gallic acid monohydrate (99%), epicatechin gallate, quercetin, quercetin-3-glucoside and quercetin-3-rutinoside, which were all supplied by Sigma–Aldrich (Chemie Check GmbH, Steinheim, Germany). The prepared solutions were stored at −20 °C and protected from light.

### 2.2. Apples Used for the Experiment

**Figure 1:**
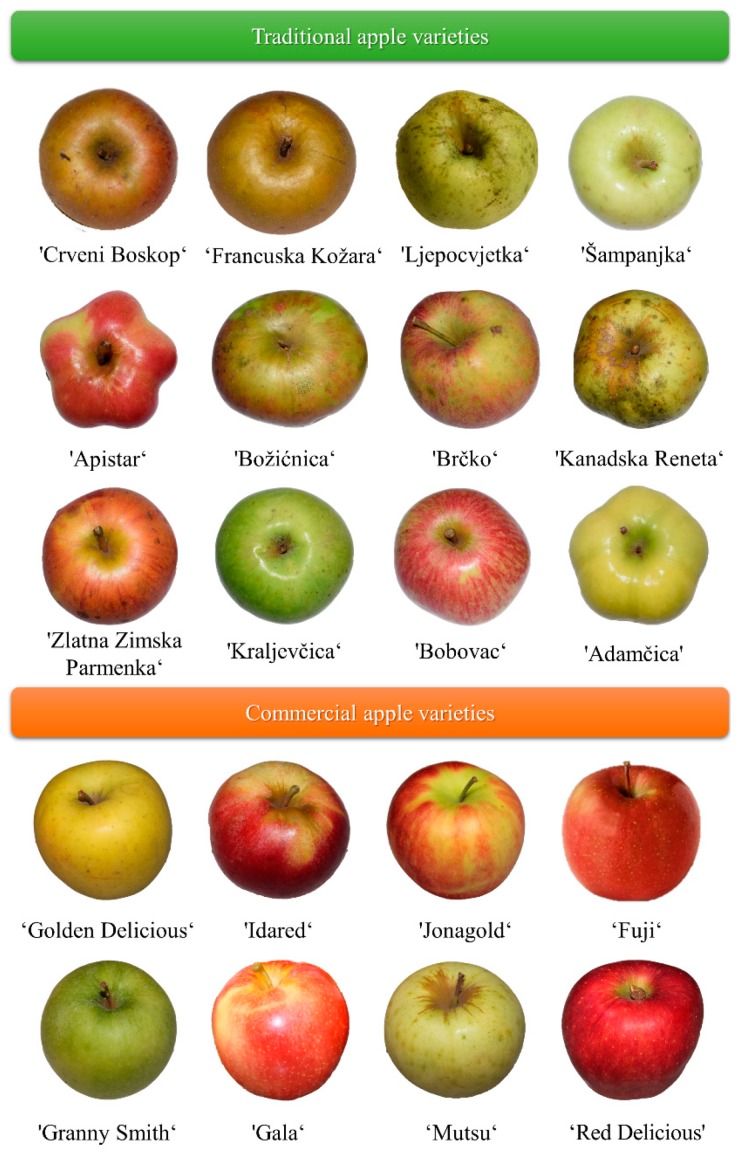
Apple varieties used in the experiment.

The traditional apple cultivars, ‘Crveni Boskop’, ‘Francuska Kožara’, ‘Ljepocvjetka’, ‘Šampanjka’, ‘Apistar’, ‘Božićnica’, ‘Brčko’, ‘Kanadska Reneta’, ‘Zlatna Zimska Parmenka’, ‘Kraljevčica’, ‘Bobovac’ and ‘Adamčica’ have been collected in the maturity stage, in October 2018 in Croatia (OPG Horvatić, Cvetkovac, Rasinja). The commercial apple cultivars, ‘Golden Delicious’, ‘Idared’, ‘Jonagold’, ‘Fuji’, ‘Granny Smith’, ‘Gala’, ‘Mutsu’ and ‘Red Delicious’ were harvested in the maturity stage, in October 2018 (OPG Pavičić, Petrijevci, Osijek, Croatia) and purchased from the local fresh market in Osijek. All the apple cultivars were subjected to sample preparation within one week. A pomologist authenticated the apple cultivars used in the study [29]. All the apples used in the experiment are shown in Figure 1. The fruit samples were peeled (2–3 mm thickness) and the apple peel was freeze-dried (Alpha LSCplus, Christ, Germany) in the temperature range from −80 to 25 °C, under 0.180 mbar. The temperature of isothermal desorption was 25 °C and the pressure was 0.060 mbar. After freeze-drying, the apple peel was pulverized in a grinder to a stabilized form and preserved in amber bottles.

### 2.3. Micro Matrix Solid-Phase Dispersion Extraction (Micro-MSPD)

**Figure 2:**
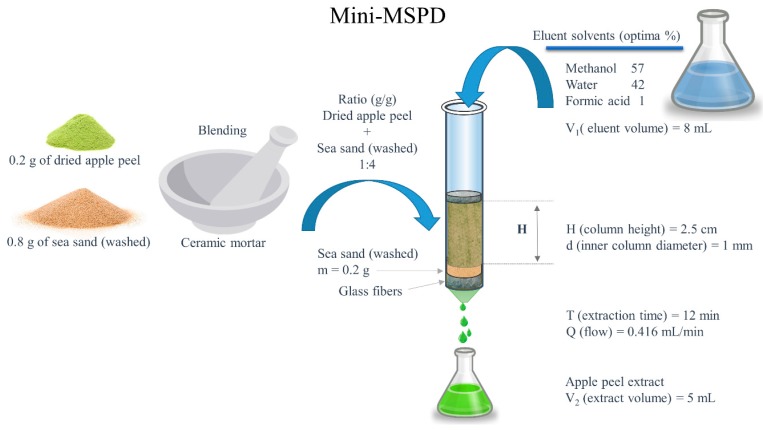
The micro matrix solid phase dispersion (micro-MSPD) procedure.

Ground freeze-dried apple peel (0.2 g) was blended with 0.8 g of washed sea sand in a mortar using a pestle until a homogeneous mixture was obtained. After blending, the mixture was transferred into a micro-MSPD column filled with glass fiber, 0.2 g of washed sea sand was placed at the bottom and glass fiber was placed on top of the sample (Figure 2). The polyphenols were eluted with mixtures of methanol:water:formic acid according to the experiments that were carried out.

In order to optimize the MSPD extraction, some preliminary experiments were conducted. The amount of methanol in the eluting solvent mixture, the mass of the dispersant and the volume of the collected extract were the variables optimized by experimental design. All the experiments were performed in duplicate and each of the obtained extracts were submitted to the analytical procedure twice. The response was evaluated in terms of the TPI content.

### 2.4. High-Performance Liquid Chromatography with Diode-Array Detectors (HPLC-PDA)

High-performance liquid chromatography was performed with the Jasco LC Net II, equipped with the AS-4150 autosampler, the PU-4180 pump and the MD-4010 PDA detector. The system was controlled with the JASCO ChromNAV Version 2.01.00 (JASCO International Co., Ltd., Tokyo, Japan). The experiments were performed on a C18 Kinetex column (150 mm × 4.6 mm, 2.6 μm; Phenomenex, Torrance, CA, USA). The mobile phase consists of A (water containing 1% formic acid) to B (methanol containing 1% formic acid). The gradient program was from 95% A to 80% in 10 min, from 80% to 70% in 5 min, from 70% to 50% in 5 min, from 50% to 0% in 5 min and isocratic for 10 min at a flow rate of 1 mL/min. Five µL of the sample was injected in duplicate onto the column kept at 50 °C.

**Figure 3:**
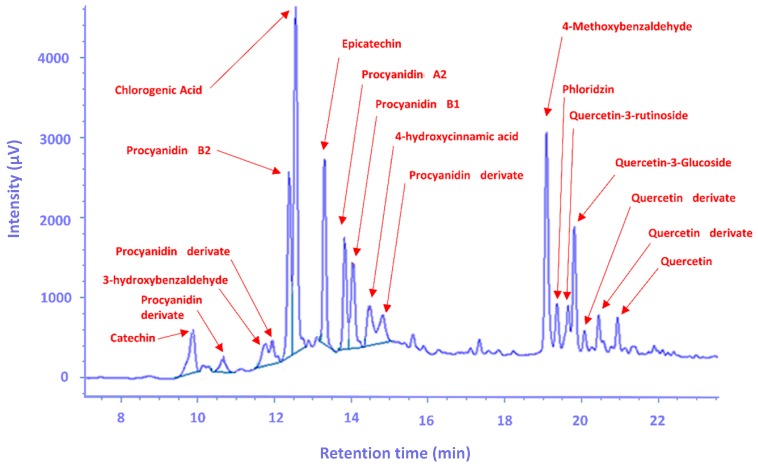
Example of an apple peal chromatogram.

The UV-Vis absorption spectra of the standards, as well as the samples, were recorded in the range of 190 to 600 nm. Polyphenols were detected at 280, 320 and 360 nm (Figure 3) and identified by the comparison of their retention times and UV-Vis spectra to those of pure standards. Quantification was performed by external standard calibration. The amount of polyphenols was expressed as mg/100 g of dw.

### 2.5. Phenolic Identification by LC-MS-MS

Identification of the major polyphenols was performed on the LC-MS/MS, in a Thermo Scientific system (San Jose, CA, USA). The system consisted of a Finnigan Surveyor™ HPLC Thermo Fisher Scientific (Madrid, Spain) and a TSQ Quantum Discovery triple stage quadrupole mass spectrometer from Thermo Fisher Scientific (Madrid, Spain), equipped with a heated electrospray ionization (HESI) source and a Thermo Scientific Hypersil Gold aQ (1.9 μm, 100 mm × 2.1 mm) column. The mobile phase was composed of (A) 0.1% formic acid/water and (B) 0.1% formic acid/methanol; with the gradient program of 0–20 min 5% B to 20% B and 20–25 min 100% B, with a flow rate of 1.0 mL/min and the column temperature of 50 °C. The column effluent was monitored by selected reaction monitoring (SRM). Polyphenols were detected in the negative and positive mode. Standards of the target phenolics were introduced into the mass spectrometer by flow injection and the collision energies of the SRM transitions were optimized for each polyphenol. Data concerning the identification of the polyphenols is shown in Table 1.

### 2.6. Determination of the Total Polyphenolic Index

The total polyphenolic index (TPI) was determined by employing the Folin–Ciocalteu reagent (FC), according to a procedure described by Singleton and Rossi [30]. The changes in the color of the radical from light blue to dark blue were measured after 30 min at 760 nm, using a UV-ViS spectrophotometer (Shimadzu UVmini-1240, Kyoto, Japan). The TP was quantified from a gallic acid calibration curve (3–20 mg/L, *r^2^* = 0.9961). The TPI was calculated and expressed as mg of a gallic acid equivalent (GAE) per g of dry weight of apple peel.

### 2.7. Determination of Antioxidant Activity

The antioxidant activity was measured using a DPPH radical, according to the modified method described by Brand-Williams, Cuvelier and Berset [31]. The reaction mixture consisted of 0.1 mL of the apple peel extract and 3.9 mL of a DPPH radical solution 0.1 mM in methanol. The changes in the color of the radical from deep violet to light yellow were measured after 30 min at 515 nm, using a UV-ViS spectrophotometer (Shimadzu UVmini-1240, Kyoto, Japan). The antiradical activity (AA) was determined using the following equation (y = 0.9548x + 0.0294; *r*^2^ = 0.9914), obtained from linear regression after plotting the A_515nm_ of known solutions of trolox against the concentration (0.1–0.9 mM). The results were expressed as millimoles of Trolox^®^ equivalents (TE) per one gram of dry apple peel (mmol TE/g dw).

### 2.8. Statistical Analysis

The experimental design applied in the optimization of the MSPD method and the subsequent data analysis were performed using the Statgraphics XV Centurion software package (Manugistics Inc., Rockville, MD, USA).

## 3. Results and Discussion

### 3.1. Preliminary Experiments

In order to set the experimental domain for the optimization of the MSPD extraction procedure, the following parameters were investigated: sample and dispersant masses at the same ratio, different sample: dispersant ratios, dispersant agent, column volume and the type of acid in the solvent system. Studying the sample (0.2 g and 0.5 g) and dispersant (0.4 g and 1.0 g) masses at the same ratio (1:2), in terms of TPI, showed that the elution time considerably decreased at low mass values and that the contact time between the sample and the solvent was more regular. For that reason, the lower masses were chosen. The sample: dispersant ratio (1:2 and 1:4.) was studied with the sample mass set to 0.2 g. In this case, significant differences in terms of TPI were observed and therefore, the sample:dispersant ratio was included as a factor in the experimental design. Regarding the nature of the dispersant, sea sand, florisil, C18, alumina and silica gel, were preliminarily assessed. This set of extractions was carried out keeping the same operative conditions (solvent type, sample and dispersant ratio and extract volume). Alumina provided the lowest responses, in terms of TPI, for the extraction of polyphenols from apple peel, while florisil, sea sand and silica gel yielded the best results. Since differences between these dispersants were not significant (*p* < 0.05) and due to the substantial difference in the price of these dispersing phases, sea sand was finally selected as the dispersant agent for MSPD. No significant differences between the columns with different volumes (2 mL and 10 mL) in terms of TPI (data not shown) were found. As the result of that, the smaller and more economical column was chosen. Finally, formic and hydrochloric acids were compared, however, there were no significant differences in terms of TPI and thus formic acid was selected, since, compared to hydrochloric acid, it is cheaper and more environmentally friendly.

### 3.2. Optimization of Micro-MSPD

Response surface methodology (RSM) was used to attain the maximum micro-MSPD extraction efficiency by optimizing the extraction parameters; factor A—extract volume (1–5 mL), factor B—proportion of methanol in the elution mixture (0%–100%) and factor C—mass of dispersant (0.6–1.1 g). A face centered central composite design 2^3^ + star was chosen. The order of the experiments was fully randomized, which enabled protection against the effects of lurking variables. The selected experimental design included seventeen experiments, which allowed evaluating the second order interactions between factors. The ANOVA results of the analysis of the obtained data are shown in Table 2 and Table 3.

**Figure 4:**
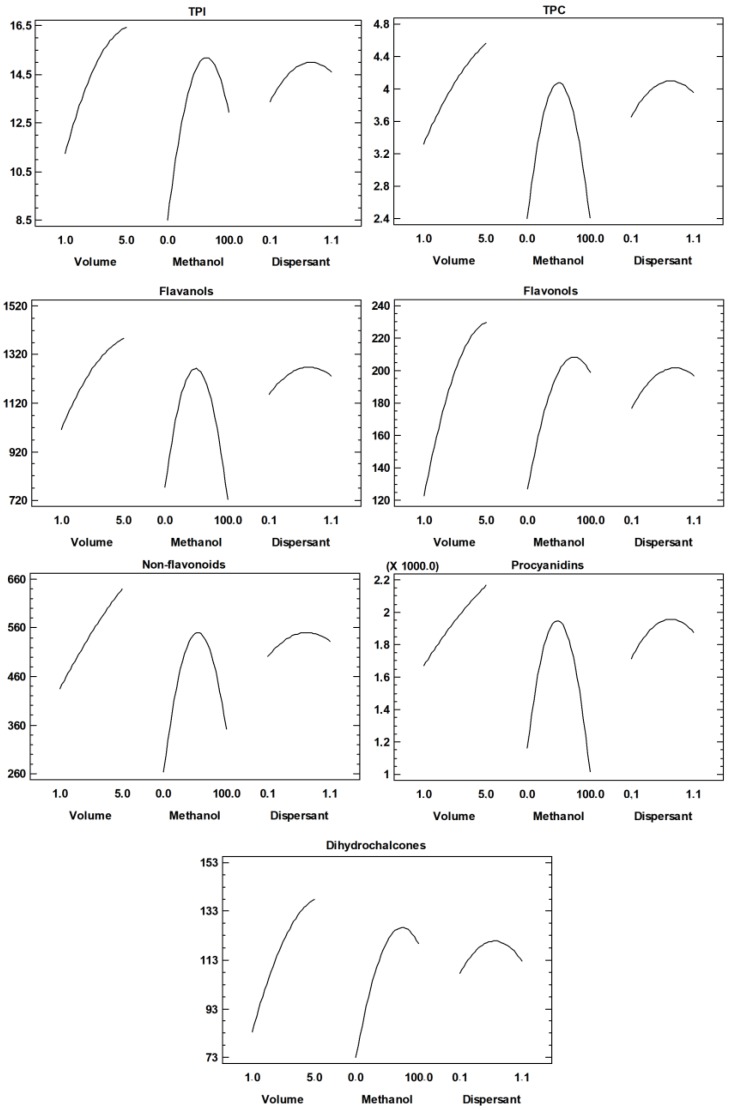
Main effect plots corresponding to the response surface methodology (RSM) design. TPI—Total polyphenolic index; TPC—Total polyphenolic content calculated from HPLC.

Regarding the extraction volume, the linear positive effect was found to have a significant influence on the investigated responses. The linear positive effect indicates that increasing the particular factor will result in greater yields of the observed response, which in this case means that increasing the volume of the extract will result in greater yields of all polyphenol groups [32]. Dispersant mass was not significant for the polyphenol yields. For the methanol percentage, the linear positive effects were also found to be statistically significant for total polyphenolic index (TPI), flavonoles, non-flavonoids and dihydrochalcones. Additionally, a significant negative quadratic effect was found for all investigated responses, which means that there is a maximum of the factor that does occur at some intermediate value (Table 3). A graphical representation of such parabolic behavior is shown in Figure 4. The observed behavior was previously reported by Álvarez-Casas et al. [33], who optimized the process of pressurized solvent extraction of polyphenolic compounds from white grape marc, as well as Assefa et al. [34], who studied the extraction of antioxidants and flavonoids from yuzu peel (*Citrus junos* Sieb ex Tanaka) and Iglesias-Carres et al. [32], who optimized the process of polyphenol extraction from sweet orange pulp. The same negative quadratic effect was found for flavonoles regarding extract volume (Table 3). The practical meaning of the interaction of a controlled factor with itself implies a quadratic model should explain the behavior of such a factor, which in practical terms implies that the optimum is somewhere in the middle of the experimental domain, not in the lowest nor the highest explored values. Moreover, the interaction between the factors, the extract volume and the methanol percentage was significant for TPI and flavonols. These results demonstrate that each individual polyphenolic compound group requires specific extraction conditions, which were expected considering different chemical nature and polarity of the polyphenolic compounds [35]. In this context, multiple response optimization was used for selecting the compromising conditions for obtaining the highest total polyphenol yields. The optimal conditions for the extraction were 5 mL of extract volume and 57% of methanol in the extraction solvent. Even though dispersant mass was not significant for the polyphenol yields, 0.8 g was chosen due to better operative conditions during the extraction procedure. The set mass of sample (0.2 g) and the mass of dispersant (0.8 g) provided the matrix/dispersant ratio of 1:4 (m/m).

### 3.3. Total Polyphenolic Index and the Antioxidant Activity of Extracts

**Figure 5:**
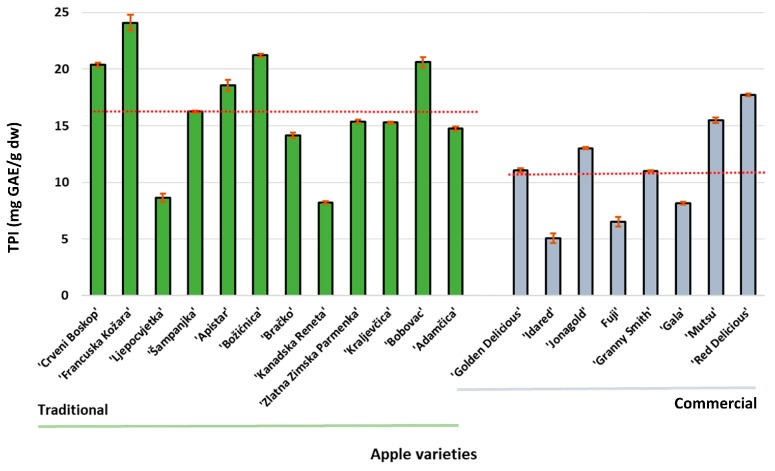
The total polyphenolic index of traditional and commercial apple varieties from Croatia. The red line is the average total polyphenolic index (TPI).

The optimized method was used for the extraction of polyphenols from twelve traditional and eight commercial apple varieties. The total polyphenolic index of investigated apple varieties is presented in Figure 5. Among the traditional apple varieties, the varieties ‘Francuska Kožara’ and ‘Red Delicious’ had the highest TPI among commercial apple varieties, 24.10 mg GAE/g dw and 17.71 mg GAE/g dw, respectively. The average values of the TPI for traditional and commercial apple varieties were 17.86 mg GAE/g dw and 13.27 mg GAE/g dw, respectively. The lower TPI for freeze-dried apple peel was reported in the literature [5,6,12]. In these studies, polyphenols were extracted from the peels of traditional and commercial apple varieties by maceration, Kschonsek et al. [12] and from commercial apple varieties by ultrasound, Lončarić et al. [5,6]; the obtained TPI values were 5.21–15.90 mg GAE/g dw and 2.88–5.72 mg GAE/g dw, respectively. Other investigations that distinguished between the TPI of old and new apple varieties are mainly conducted without separately analyzing the peel and the flesh and those showed a higher TPI content of traditional varieties compared to commercial apple varieties [2,36,37,38].

**Figure 6:**
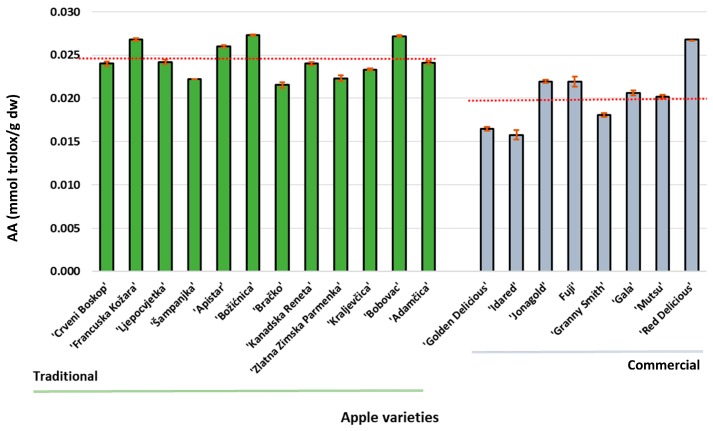
The antioxidant activity of traditional and commercial apple varieties from Croatia. The red line is the average antioxidative activity (AA).

The results of the antioxidant activity of the peel extracts are shown in Figure 6. The correlation between the total polyphenols and the antioxidant activity is significant (*r* = 0.7359) at a confidence level of *p* = 0.05. However, there was no statistical difference (*p* < 0.05) between the varieties ‘Francuska Kožara’, ‘Božićnica’, ‘Bobovec’ and ‘Red Delicious’. The average AA values of traditional and commercial apples were 0.024 mmol Trolox/g dw and 0.020 mmol Trolox/g dw, respectively. These results were comparable to previously reported study results [12,17].

### 3.4. Individual Polyphenolic Profile of Extracts

The content of individual polyphenols is shown in Table 4, Table 5, Table 6 and Table 7. The contents of catechin, epicatechin, phloridzin, procyanidins, flavonoles and chlorogenic acid were in the range of those reported by Wojdylo et al. [39], who studied 67 new and old apple varieties. The contribution of catechin and epicatechin in traditional and commercial apple varieties was 9.9% and 11.5% of total polyphenols, respectively. In both cases, epicatechin was the predominant polyphenol. The contribution of phloridzin was 4.8% in traditional and 2.4% in commercial apple varieties. The varieties ‘Apistar’, ‘Bobovac’ and ‘Božićnica’ had the highest content of catechin (512.36 µg/g dw), epicatechin (1317.78 µg/g dw) and phloridzin (577.58 µg/g dw), respectively (Table 4).

The contribution of procyanidins was 26.9% in traditional and 46.2% in commercial varieties, with procyanidin B2 as a major compound in this group. The variety ‘Bobovac’ had the highest amount of procyanidin A2 (330.75 µg/g dw) and B2 (1574.71 µg/g dw); ‘Jonagold’ had the highest amount of procyanidin B1 (738.95 µg/g dw) and considering procyanidin derivatives, the variety ‘Adamčica’ had the highest amount 517.87 µg/g dw (*p* < 0.05; Table 5). The higher amount of flavanols and phloridzin in traditional varieties, compared to commercial apple varieties, was also reported by Kschonsek et al. [11]. However, the amounts of catechin, epicatechin and procyanidins were significantly higher than the amounts reported by the same author earlier. In this study, polyphenols from 15 traditional and commercial freeze-dried apple peels were extracted by using the following extraction procedure: 75% methanol with 1 M hydrochloric acid were used as solvent and the samples were heated in a water bath for 30 min at 37 °C [12]. The amount of phloridzin was in the range, with Goldparmäne variety having the higher amount of phloridzin (638 µg/g dw) than reported in this study. These results are expected given the used solvent. Herbey applied the optimum methanol proportion in the extraction solvent, calculated by RSM for flavanols and dihydrochalcones, which was 47% and 80%, respectively. A similar trend was reported by Jakobek et al. [40], who studied the influence of various ratios of methanol and water for the extraction of polyphenols from apple flesh and peel based on ultrasound-assisted maceration.

The contribution of flavonols was only 26.8% in traditional and 20.7% in commercial varieties, which is significantly lower than reported in other studies, 72.2% by Kschonsek et al. [12] and 74% by Jakobek and Baron [2]. The reason for the lower amount of the extracted flavonols could be the lower proportion of methanol in the extraction solvent compared to those used in the above studies. Results presented in Figure 3 imply that two groups of polyphenolic compounds are separated by their polarity. The optimum methanol proportion in the extraction solvent calculated by RSM for flavonols applied in this study was 86%. Another reason for the lower amount of flavonols could be the environmental conditions. Contrary to the other groups of polyphenolic compounds, flavonols are more susceptible to environmental changes, especially to light and temperature changes [41]. The traditional apple variety ‘Bobovac’ had the highest content of quercetin (270.48 µg/g dw), quercetin-3-glucoside (178.63 µg/g dw) and quercetin derivative-1 (390.52 µg/g dw; *p* < 0.05; Table 6). Whereas, ‘Zlatna Zimska Parmenka’ (2383.28 µg/g dw) and ‘Božićnica’ (2244.46 µg/g dw) had the highest content of quercetin-3-rutinoside, which was also the major polyphenol contributing to total flavonol percentage (*p* < 0.05). The high content of the quercetin derivative found in the peel could be the result of hydrolysis during the extraction [42,43].

Finally, the contribution of non-flavonoids was 28.6% in traditional and 15.9% in commercial apple varieties. The variety ‘Ljepocvjetka’ had the highest content of chlorogenic acid (2334.11 µg/g dw; *p* < 0.05; Table 7). Chlorogenic acid was the most abundant compound in this group of polyphenols, considering both traditional and commercial apple varieties. As can be seen from the results, traditional apple varieties are dominated by non-flavonoids (chlorogenic acid) and commercial apple varieties are dominated by flavanols (procyanidins). Other authors also observed this difference among different apple varieties [10,37,40].

Many studies emphasize the health-promoting effects of different polyphenols. Some of these polyphenols were extracted in this study in great abundance (≥1000 µg/g dw). First of them is epicatechin (>1300 µg/g dw), it was reported that the consumption of epicatechin reduces blood glucose levels in diabetic patients and has an anticancer effect, which was attributed to its antioxidant properties, as well as antiangiogenic and direct cytotoxicity to cancer cells [44]. The second one is procyanidin B2 (>1500 µg/g dw). Studies have shown that proanthocyanidins help in protecting the body from sun damage, improving the flexibility of heart tissues, improving blood circulation by strengthening blood vessels and even improving vision [45]. Furthermore, quercetin-3-rutinoside (>2300 µg/g dw), Que3glu, has been used conventionally as an antimicrobial, antifungal and anti-allergic agent and certain research has shown pharmacological benefits for the treatment of various chronic diseases such as cancer, diabetes, hypertension and hypercholesterolemia [46]. Finally, chlorogenic acid (>2300 µg/g dw), CA, is also an important and biologically active dietary polyphenol, playing several important and therapeutic roles, such as antioxidant activity, antibacterial, hepatoprotective, cardioprotective roles, etc. In addition, it has been found that CA could modulate lipid metabolism and glucose in both genetically and metabolically related health disorders [47].

## 4. Conclusions

Reportedly, this is the first use of micro-matrix solid-phase dispersion optimized by RSM for the extraction of polyphenols from apple peel. Furthermore, optimized micro-MSPD provides high yields of total polyphenols, requiring low solvent consumption and short time when compared to standard extraction methods. There are eighteen polyphenols positively identified by LC-MS and quantified by HPLC-DAD. The most abundant polyphenol in traditional apple varieties was chlorogenic acid and in commercial apple varieties it was procyanidin B2. However, the traditional apple varieties had a higher content of all identified polyphenolic compounds (except PB1 ‘Jonagold’), resulting in a higher antioxidant activity compared to commercial apple varieties. Accordingly, the recommendation should be to consume traditional apple varieties with peel, preferably varieties similar to ‘Bobovac’, in order to increase the daily polyphenol intake.

## Figures and Tables

**Table 1:** Identities, retention times and MS/MS experimental parameters for the target compounds.

Phenolic Compound	Molecular Formula	[M − H]^−^ (*m*/*z*) ^a^	MS/MS (*m*/*z*) ^b^	Collision Energy	Rt (min) HPLC ^c^
Catechin ^+^	C_15_H_14_O_6_	289.006	245.020 203.115	17 22	9.53
Epicatechin ^+^	C_15_H_14_O_6_	289.006	245.020 203.115	17 22	13.35
Phloridzin	C_21_H_24_O_10_	273.100	166.800	35	19.20
Procyanidin A2 ^+^	C_30_H_24_O_12_	577.090	287.002 136.988 425.081 437.078	32 62 13 16	13.56
Procyanidin B1	C_30_H_26_O_12_	577.033	407.066 288.931 424.977	26 25 26	14.24
Procyanidin B2	C_30_H_26_O_12_	577.033	407.066 288.931 424.977	26 25 26	12.15
Procyanidin derivative 1	C_30_H_26_O_12_	577.033	−	−	10.56
Procyanidin derivative 2	C_30_H_26_O_12_	577.033	−	−	11.76
Procyanidin derivative 3^+^	C_30_H_26_O_12_	577.090	−	−	14.94
Quercetin ^+^	C_15_H_10_O_7_	303.098	229.106 153.046	28 33	21.89
Quercetin-3-glucoside ^+^	C_21_H_20_O_12_	465.103	461.500 302.966	14 18	19.45
Quercetin-3-rutinoside	C_27_H_30_O_16_	609.182	270.917 178.876 300.013	56 44 37	19.31
Quercetin derivative 1 ^+^	C_n_H_n_O_n_	319.000	−	−	19.99
Quercetin derivative 2 ^+^	C_n_H_n_O_n_	285.078	−	−	20.46
3-Hydroxybenzaldehyde ^+^	C_7_H_6_O_2_	121.016	93.056 92.054	20 23	11.52
4-Hydroxycinnamic acid ^+^	C_9_H_8_O_3_	163.016	119.072 163.016 163.016	18 37 38	14.70
Chlorogenic acid ^+^	C_16_H_18_O_9_	353.000	191.074 85.090 93.073	22 43 45	12.38
4-Methoxybenzaldehyde ^+^	C_8_H_8_O_2_	136.977	109.058 77.056 94.041	12 23 18	18.52

^a^: [M − H]^−^ = Molecular ion weight. ^b^: MS/MS = Fragmented phenolic molecular weight. ^c^: −Rt = retention time (min) from the Jasco LC Net II HPLC. ^+^: Compounds detected in positive mode [M + H]^+^.

**Table 2:** The *F* and *p* values for the factors obtained in the analysis of variance for the response surface design of three factors at two levels.

	Factors					
	A: Extract Volume		B: % Methanol		C: Mass of Dispersant	
	*F*	*p*	*F*	*p*	*F*	*p*
TPI	71.98	0.00 ^a^	51.89	0.00 ^a^	3.93	0.09
TPC	89.43	0.00 ^a^	0.00	0.95	5.12	0.06
Flavanols	104.68	0.00 ^a^	1.85	0.22	4.41	0.07
Flavonols	159.13	0.00 ^a^	70.48	0.00 ^a^	5.55	0.05
Non-flavonoids	75.96	0.00 ^a^	14.12	0.01 ^a^	1.83	0.22
Procyanidins	46.91	0.00 ^a^	4.20	0.08	5.03	0.06
Dihydrochalcones	75.61	0.00 ^a^	55.11	0.00 ^a^	0.66	0.44

TPI—Total polyphenolic index; TPC—Total polyphenolic content (HPLC) (mg/g dw); ^a^: Significant effects for a confidence level of 95%.

**Table 3:** The *F* and *p* values for the factor interactions obtained in the analysis of variance for the response surface design of three factors at two levels.

	Interactions											
	AA		AB		AC		BB		BC		CC	
	*F*	*p*	*F*	*p*	*F*	*p*	*F*	*p*	*F*	*p*	*F*	*p*
TPI	3.27	0.11	6.56	0.04 ^a^	0.05	0.83	50.06	0.00 ^a^	0.53	0.49	2.40	0.17
TPC	1.15	0.32	0.00	0.98	0.05	0.83	175.30	0.00 ^a^	0.28	0.61	4.70	0.07
Flavanols	3.17	0.12	3.31	0.11	0.28	0.61	213.01	0.00 ^a^	0.10	0.76	3.75	0.09
Flavonols	8.09	0.03^a^	5.98	0.04 ^a^	0.18	0.69	20.00	0.00 ^a^	3.01	0.13	2.44	0.16
Non-flavonoids	0.21	0.66	4.14	0.08	0.02	0.90	111.32	0.00 ^a^	0.47	0.52	1.93	0.21
Procyanidins	0.18	0.69	0.00	0.97	0.05	0.84	150.26	0.00 ^a^	0.25	0.63	4.63	0.07
Dihydrochalcones	2.71	0.14	0.46	0.52	0.51	0.50	15.98	0.01 ^a^	5.05	0.06	3.04	0.12

TPI—Total polyphenolic index; TPC—Total polyphenolic content (HPLC; mg/g dw). A: extract volume; B: % methanol; C: mass of dispersant; ^a^: Significant effects for a confidence level of 95%.

**Table 4:** The content of flavanols and dihydrochalcones (µg/g dw ^a^) in the apple peels.

		Catechin	Epicatechin	Phloridzin
Traditional	‘Crveni Boskop’	74.97 ± 1.67 ^g,^*	219.52 ± 10.25 ^m^	77.48 ± 4.50 ^j^
	‘Francuska Kožara’	n.d.	311.03 ± 4.88 ^j^	87.52 ± 3.45 ^i^
	‘Ljepocvjetka’	n.d.	371.00 ± 22.67 ^h^	212.86 ± 6.83 ^e^
	‘Šampanjka’	n.d.	167.71 ± 10.70 ^n^	556.20 ± 15.90 ^b^
	‘Apistar’	512.36 ± 12.20 ^a^	1194.72 ± 21.41 ^b^	447.79 ± 10.08 ^c^
	‘Božicnica’	172.39 ± 1.84 ^d^	504.37 ± 14.17 ^f^	577.58 ± 8.80 ^a^
	‘Brčko’	72.93 ± 3.91 ^g^	492.17 ± 12.28 ^f^	111.13 ± 6.95 ^g^
	‘Kanadska Reneta’	117.79 ± 2.19 ^f^	565.03 ± 20.47 ^e^	69.99 ± 2.14 ^j^
	‘Zlatna Zimska Parmenka’	150.46 ± 2.00 ^e^	278.73 ± 3.07 ^k^	386.23 ± 16.98 ^d^
	‘Kraljevčica’	303.33 ± 17.18 ^b^	663.72 ± 31.99 ^c^	n.d.
	‘Bobovac’	186.46 ± 1.12 ^c^	1317.78 ± 10.80 ^a^	442.42 ± 9.35 ^c^
	‘Adamčica’	165.86 ± 3.56 ^d^	627.69 ± 7.80 ^d^	151.40 ± 4.30 ^f^
Commercial	‘Golden Delicious’	n.d.	260.33 ± 3.71 ^k,l^	72.88 ± 0.35 ^j^
	‘Idared’	n.d.	144.96 ± 1.94 ^o^	44.15 ± 0.66 ^k^
	‘Jonagold’	n.d.	320.39 ± 4.90 ^j^	69.15 ± 1.51 ^j^
	‘Fuji’	27.28 ± 2.28 ^i^	256.25 ± 8.83 ^l^	38.22 ± 2.39 ^k^
	‘Granny Smith’	47.45 ± 1.17 ^h^	201.72 ± 5.61 ^m^	98.35 ± 2.87 ^h^
	‘Gala’	n.d.	413.49 ± 20.85 ^g^	73.28 ± 2.77 ^j^
	‘Mutsu’	n.d.	344.02 ± 5.12 ^i^	45.89 ± 0.79 ^k^
	‘Red Delicious’	n.d.	612.49 ± 9.26 ^d^	n.d.

^a^: Dry weight. Mean ± SD based on two extracts each measured twice (*n* = 4), * *p* < 0.05. Different letters in each column indicate significant differences at 95% confidence level as obtained by the LSD test. n.d.—not detected.

**Table 5:** The content of procyanidins (µg/g dw ^a^) in the apple peels.

	PA2	PB1		PB2	PD1	PD2	PD3
Traditional	‘Crveni Boskop’	154.25 ± 5.20 ^i,j,^*	n.d.	663.59 ± 23.75 ^h^	n.d.	n.d.	n.d.
	‘Francuska Kožara’	193.61 ± 3.29 ^f,g^	n.d.	999.41 ± 31.06 ^e^	n.d.	n.d.	n.d.
	‘Ljepocvjetka’	240.61 ± 11.25 ^d^	219.36 ± 10.57 ^f^	871.20 ± 53.26 ^f^	n.d.	n.d.	n.d.
	‘Šampanjka’	147.81 ± 5.58 ^j^	212.99 ± 8.18 ^f^	451.35 ± 22.61 ^j^	n.d.	n.d.	n.d.
	‘Apistar’	277.02 ± 4.38 ^b^	n.d.	1402.37± 32.48 ^b^	n.d.	n.d.	n.d.
	‘Božicnica’	278.92 ± 4.03 ^b^	n.d.	1201.01± 27.35 ^c^	n.d.	n.d.	n.d.
	‘Brčko’	187.96 ± 6.45 ^g^	699.35 ± 22.83 ^b^	727.55± 30.99 ^g^	116.37 ± 2.36 ^a^	n.d.	182.93 ± 5.51 ^d^
	‘Kanadska Reneta’	266.02 ± 3.94 ^c^	328.83 ± 10.05 ^d^	1235.70± 37.19 ^c^	n.d.	n.d.	303.33 ± 3.77 ^b^
	‘Zlatna Zimska Parmenka’	168.97 ± 1.45 ^h^	n.d.	618.42 ± 7.59 ^i^	n.d.	n.d.	240.92 ± 3.65 ^c^
	‘Kraljevčica’	211.20 ± 5.61 ^e^	n.d.	714.86 ± 24.75 ^g^	n.d.	n.d.	352.99 ± 14.65 ^a^
	‘Bobovac’	330.75 ± 26.78 ^a^	n.d.	1574.71 ± 19.79 ^a^	n.d.	n.d.	n.d.
	‘Adamčica’	231.90 ± 0.97 ^d^	248.71 ± 4.93 ^e^	986.56 ± 20.56 ^e^	106.29 ± 1.42 ^b^	177.42 ± 4.42 ^a^	234.16 ± 2.58^c^
Commercial	‘Golden Delicious’	n.d.	183.70 ± 3.74 ^g^	n.d.	101.09 ± 0.30 ^d^	129.42 ± 0.75 ^b^	173.47 ± 1.95 ^e^
	‘Idared’	134.13 ± 3.20 ^k^	352.11 ± 1.61 ^c^	292.97 ± 6.09 ^k^	93.85 ± 3.69 ^e^	114.84 ± 1.16 ^d^	143.77 ± 2.06^f^
	‘Jonagold’	160.48 ± 8.37 ^h,i^	738.95 ± 34.27 ^a^	452.53 ± 28.03 ^j^	n.d.	120.42 ± 1.55 ^c^	173.32 ± 3.75 ^e^
	‘Fuji’	166.82 ± 2.17 ^h^	n.d.	453.70 ± 66.42 ^j^	n.d.	96.94 ± 4.92 ^e^	147.34 ± 7.45 ^f^
	‘Granny Smith’	136.10 ± 3.12 ^k^	101.37 ± 12.46 ^j^	476.52 ± 6.09 ^j^	103.26 ± 0.76 ^c^	n.d.	130.18 ± 15.40 ^g^
	‘Gala’	183.24 ± 6.22 ^g^	161.71 ± 3.40 ^h^	618.62 ± 2.28 ^i^	n.d.	n.d.	n.d.
	‘Mutsu’	200.87 ± 2.15 ^e,f^	136.88 ± 2.03 ^i^	837.11 ± 12.84 ^f^	n.d.	n.d.	n.d.
	‘Red Delicious’	105.29 ± 0.60 ^l^	n.d.	1112.70 ± 28.48 ^d^	n.d.	n.d.	n.d.

^a^: Dry weight. Mean ± SD based on two extracts each measured twice (*n* = 4). * *p* < 0.05. Different letters in each column indicate significant differences at 95% confidence level as obtained by the LSD test. PA2—Procyanidin A2; PB1—Procyanidin B1; PB2–Procyanidin B2; PD1–Procyanidin derivative 1; PD2–Procyanidin derivative 2; PD3—Procyanidin derivative 3.

**Table 6:** The content of flavonols (µg/g dw ^a^) in apple peels.

		Que	Que3glu	Que3rut	QueD1	QueD2
Traditional	‘Crveni Boskop’	49.01 ± 3.45 ^m^	52.67 ± 3.990 ^k^	n.d.	96.05 ± 9.01 ^g^	27.47 ± 0.99 ^h^
	‘Francuska Kožara’	n.d.	56.00 ± 1.79^jk^	63.95 ± 1.41 ^e,f^	133.05 ± 4.93 ^f^	47.72 ± 0.93 ^g^
	‘Ljepocvjetka’	209.31 ± 7.98 ^b^	108.88 ± 3.77 ^f^	n.d.	232.63 ± 20.93 ^e^	n.d.
	‘Šampanjka’	115.93 ± 4.80 ^f,g^	135.31 ± 5.36 ^d^	991.43 ± 62.49 ^c^	245.62 ± 10.72 ^e^	n.d.
	‘Apistar’	119.73 ± 1.98 ^e,f^	141.01 ± 2.92 ^c^	956.56 ± 24.61 ^c^	341.83 ± 7.49 ^b^	n.d.
	‘Božicnica’	121.72 ± 1.08 ^e^	153.50 ± 2.08 ^b^	2244.46 ± 71.91 ^a^	320.13 ± 4.28 ^c^	508.82 ± 21.81 ^a^
	‘Brčko’	65.73 ± 3.14 ^k^	63.47 ± 0.98 ^i^	234.66 ± 10.35 ^d,e,f^	131.51 ± 6.38 ^f^	n.d.
	‘Kanadska Reneta’	79.51 ± 1.68 ^i^	57.69 ± 1.90 ^j^	858.81 ± 5.77 ^c^	85.57 ± 3.17 ^g,h^	225.08 ± 13.04 ^d^
	‘Zlatna Zimska Parmenka’	54.31 ± 3.05 ^l^	75.32 ± 1.50 ^g^	2383.28 ± 77.13 ^a^	133.74 ± 9.20 ^f^	n.d.
	‘Kraljevčica’	119.97 ± 5.16 ^e,f^	129.93 ± 6.30 ^e^	1621.28 ± 57.42 ^b^	285.03 ± 14.59 ^d^	459.16 ± 20.80 ^b^
	‘Bobovac’	270.48 ± 1.56 ^a^	178.63 ± 1.73 ^a^	1632.51 ± 939.02 ^b^	390.52 ± 40.41 ^a^	360.78 ± 22.48 ^c^
	‘Adamčica’	56.36 ± 1.83 ^l^	46.41 ± 1.42 ^l^	n.d.	75.78 ± 2.51 ^h^	n.d.
Commercial	‘Golden Delicious’	111.60 ± 1.77 ^g^	55.36 ± 1.07 ^j,k^	296.53 ± 4.90 ^d,e,f^	n.d.	65.21 ± 0.89 ^f^
	‘Idared’	46.23 ± 1.48 ^m^	n.d.	90.09 ± 1.83 ^e,f^	88.33 ± 0.67 ^g,h^	n.d.
	‘Jonagold’	148.15 ± 5.92 ^d^	70.76 ± 3.23 ^h^	413.42 ± 12.98 ^d^	133.05 ± 6.23 ^f^	89.05 ± 4.12 ^e^
	‘Fuji’	173.32 ± 3.69 ^c^	39.68 ± 3.62 ^m^	316.20 ± 56.38 ^d,e^	79.31 ± 9.30 ^g,h^	70.54 ± 9.18 ^f^
	‘Granny Smith’	76.61 ± 0.85 ^i,j^	57.10 ± 0.34 ^j^	n.d.	76.51 ± 3.70 ^h^	n.d.
	‘Gala’	71.98 ± 3.85 ^j^	56.04 ± 3.52 ^j,k^	n.d.	121.98 ± 8.24 ^f^	n.d.
	‘Mutsu’	106.12 ± 4.28 ^h^	46.25 ± 1.30 ^l^	260.07 ± 5.64 ^d,e,f^	85.77 ± 2.994 ^g,h^	72.37 ± 0.71 ^f^
	‘Red Delicious’	61.71 ± 0.82 ^k^	64.64 ± 0.87 ^i^	n.d.	120.06 ± 1.95 ^f^	225.16 ± 3.66 ^d^

^a^: Dry weight. Mean ± SD based on two extracts each measured twice (*n* = 4), * *p* < 0.05. Different letters in each column indicate significant differences at 95% confidence level as obtained by the LSD test. Que—quercetin; Que3glu–quercetin-3-glucoside; Que3rut—quercetin-3-rutinoside; Qued1—quercetin derivative 1; Qued2—quercetin derivative 2; n.d.—not detected.

**Table 7:** The content of non-flavonoids (µg/g dw ^a^) in apple peels.

		3-Hba	4-Hca	CA	4-Mba
Traditional	‘Crveni Boskop’	n.d.	236.76 ± 18.26 ^d^	877.22 ± 34.89 ^g^	69.17 ± 0.94 ^i^
	‘Francuska Kožara’	n.d.	408.77 ± 11.90 ^a^	2102.58 ± 57.64 ^b^	20.05 ± 6.51 ^n^
	‘Ljepocvjetka’	n.d.	199.82 ± 11.68 ^e^	2334.11 ± 33.27 ^a^	219.67 ± 1.85 ^d^
	‘Šampanjka’	n.d.	93.92 ± 6.76 ^i^	1952.01 ± 71.40 ^c^	336.19 ± 5.70 ^b^
	‘Apistar’	183.06 ± 1.66 ^c^	241.14 ± 4.82 ^d^	203.66 ± 4.75 ^j^	280.08 ± 5.34 ^c^
	‘Božicnica’	n.d.	372.73 ± 5.48 ^b^	1212.62 ± 38.03 ^e^	218.21 ± 2.38 ^d^
	‘Brčko’	n.d.	n.d.	112.79 ± 3.54 ^l^	145.65 ± 1.89 ^g^
	‘Kanadska Reneta’	148.70 ± 3.05 ^d^	113.10 ± 0.95 ^h^	903.08 ± 25.67 ^g^	33.05 ± 1.75 ^m^
	‘Zlatna Zimska Parmenka’	n.d.	n.d.	1175.78 ± 10.19 ^e^	153.34 ± 2.71 ^f^
	‘Kraljevčica’	213.46 ± 8.83 ^b^	282.21 ± 15.41 ^c^	172.26 ± 11.83 ^j,k^	182.07 ± 2.21 ^e^
	‘Bobovac’	297.01 ± 13.94 ^a^	n.d.	1646.92 ± 12.47 ^d^	479.80 ± 2.95 ^a^
	‘Adamčica’	n.d.	n.d.	1021.78 ± 47.19 ^f^	49.11 ± 3.42 ^l^
Commercial	‘Golden Delicious’	n.d.	n.d.	378.55 ± 5.25 ^h^	66.96 ± 1.99 ^i,j^
	‘Idared’	n.d.	n.d.	330.06 ± 3.91 ^i^	31.60 ± 2.29 ^m^
	‘Jonagold’	n.d.	n.d.	207.01 ± 3.70 ^j^	96.16 ± 3.21 ^h^
	‘Fuji’	n.d.	n.d.	156.21 ± 30.10 ^k^	30.55 ± 4.49 ^m^
	‘Granny Smith’	114.71 ± 1.72 ^e^	n.d.	n.d.	57.99 ± 1.63 ^k^
	‘Gala’	n.d.	165.14 ± 8.03 ^g^	308.10 ± 4.52 ^i^	66.07 ± 0.74 ^j^
	‘Mutsu’	n.d.	177.37 ± 6.57 ^f^	375.92 ± 7.65 ^h^	22.15 ± 3.10 ^n^
	‘Red Delicious’	n.d.	277.63 ± 4.22 ^c^	n.d.	48.90 ± 3.75 ^l^

^a^: Dry weight. Mean ± SD based on two extracts each measured twice (*n* = 4), * *p* < 0.05. Different letters in each column indicate significant differences at 95% confidence level as obtained by the LSD test. 3Hba—3-Hydroxybenzaldehyde; 4Hca—4-Hydroxycinnamic acid; CA Chlorogenic acid; 4Mba—4-Methoxybenzaldehyde. n.d.—not detected.
